# Implementing practice guidelines: easier said than done

**DOI:** 10.1186/2045-4015-3-20

**Published:** 2014-06-20

**Authors:** Saul N Weingart

**Affiliations:** 1 Tufts Medical Center, 800 Washington St., Boston, MA 02111, USA

## Abstract

Implementation of practice guidelines is a beguilingly complex activity that requires attention to the task of clinicians, the constraints they face, and the social practice of medicine. Local clinical opinion leaders can accelerate the pace of change by encouraging early adoption and modeling new practices. “Tough love” approaches to guideline adoption have a role in raising the salience of the safe practice. However, successful implementation requires a healthy respect for the challenge of enlisting frontline practitioners in integrating changes into the practice of active clinicians. The implementation of guideline-based practices for aseptic technique in neuraxial analgesia at four Israeli hospitals illustrates the challenges and opportunities associated with changing physician practice.

## 

Among health policy makers and hospital administrators, few things are potentially as rewarding and at times as frustrating as changing physician behavior. Leaders are often charged with initiatives such as introducing an electronic health record, developing a novel clinical protocol, adopting measures to meet a regulatory requirement, or implementing a practice guideline. In each case, getting physicians to do something different is no simple matter.

And why should it be so? We train doctors to think independently, applying expert knowledge based on science and clinical experience. We then place them in demanding, high-risk settings where there is little margin for error. Physicians develop habits of practice that reflect their training, their subsequent experience, patient populations they serve, and local conditions under which they work. To change professional practice, change-agents need to communicate the scientific basis for the change, help clinicians to appreciate the need and value of the change relative to current practice, cultivate clinicians’ willingness to proceed, provide the tools and resources to make the change, and create conditions that make it a lasting success.

## Half-full or half-empty?

A case study in practice change, Ioscovich et al. report in the *Journal* a survey of self-reported changes in aseptic technique during neuraxial analgesia for labor at four Israeli medical centers before and after the dissemination of international guidelines [1]. The guidelines call for several safe practices during this procedure: hand hygiene, removal of jewelry, and use of a surgical mask, cap, and sterile gown. Surveying attending anesthesiologists, anesthesia residents, and supervised, post-residency physicians at baseline in 2006 and again 2009 after guideline dissemination, the authors identified significant improvements in compliance across all clinician groups and hospitals. For the composite endpoint of “always” using hand disinfection and a surgical mask to perform the procedure, self-reported compliance increased from 33% to 58%, a statistically significant difference.

The results are heartening, indicating widespread adoption of consensus-based safety practices in anesthesia. The results are good news for patients and for health policy makers. Respondents reported fewer infectious complications at follow up compared to baseline, a finding that suggests a relationship between practice change and key clinical outcomes.

Despite the apparent success of the neuraxial anesthesia guideline implementation -- subject to the inherent limitations of self-reporting studies [2] -- one wonders why Israeli anesthesiologists have not moved even more quickly and effectively to adopt the new guidelines. If the guidelines represent a consensus in the profession about the practice of neuraxial anesthesia, why doesn’t everyone use them all the time? Should we be satisfied with the progress to date, or disappointed with our failure to achieve full compliance?

## Implementation challenges

A closer look at the data suggests differential uptake among practitioners and across hospitals, hinting at the complexities inherent in the use of guidelines to drive practice change. Residents were substantially more likely to comply with the recommended elements of aseptic technique than attendings or non-resident physicians in both 2009 and 2006, although compliance increased in all groups. In addition, the busiest clinicians were least likely to change practice compared to their less active peers. While the overall direction was positive, a more granular view suggests inconsistent uptake and spread of practice guidelines both within and across the study hospitals. How can we make sense of this heterogeneity, and how does it inform efforts to change physician behavior?

Students of implementation science have long recognized that dissemination of innovation is a complex phenomenon. Early studies by Rogers, drawing initially on the spread of innovation in agriculture, identified attributes of innovations that speed their adoption [3]. Successful innovations must demonstrate relative advantage of the new practice over the old one. They must be compatible with the beliefs or values of practitioners. They must be simple enough for individuals to understand and incorporate into existing practice. They must be easy for the practitioner to try out. And it must be possible to observe how others have begun to use them.

Beyond attributes of the guideline itself, implementation scientists describe additional factors that mediate uptake and adherence. Davis and Taylor-Vaisey, in a systematic review of the literature, observed that the characteristics of health professionals, practice setting, incentives, regulatory environment, and patient factors played a role in translating guidelines into practice [4]. In another systematic review, Cabana and colleagues found that lack of awareness of the guideline’s existence or familiarity with its details are common barriers to adherence [5]. Even among physicians who are knowledgeable about the guidelines, individuals may disagree with the recommendations, doubt their efficacy, or lack motivation to change longstanding habits of practice (so-called practice “intertia”). Physicians may also find the guidelines impractical to implement due to external factors such as limited time, resources, or reimbursement. Since most guidelines are based on expert opinion rather than the findings of randomized, controlled trials, clinicians are increasingly skeptical about potential biases affecting the recommendations of consensus panels [6].

These principles of implementation science help us to interpret the findings of the Ioscovich study. Resident physicians were much more likely than other practitioners to follow the new guidelines. Junior physicians are often early adopters because they have less practice inertia to overcome, and more readily incorporate new, evidence-based guidelines into their practice. This phenomenon is seen also in studies of electronic medication safety alerts, where junior physicians were more likely to accept practice-changing advice than established practitioners [7]. Trainees are generally more receptive to practice guidance in part because they have less confidence in their base of experience and clinical judgment, compared to senior physicians.

The poor rate of guideline compliance among the most active clinicians suggests that experienced providers value their practice habits, clinical judgment, and professional autonomy over an externally imposed guideline. Alternatively, the busiest clinicians have the least opportunity to study new recommendations and to integrate these activities into their practices. In addition, busy clinicians may have the most to lose in productivity during an adaptation or transition process, and administrators may be loath to commit additional resources to facilitate practice changes in high-performing units. Policy makers and administrators struggle with how to engage the most active clinicians in new practices, since their engagement is essential to successful implementation and failure to do so may be demoralizing for colleagues who follow the rules.

## Improving compliance

Strategies for disseminating clinical guidelines often rely exclusively on information and education. These approaches are generally weak interventions, less effective than initiatives that audit and provide feedback to individual clinicians regarding their own performance [4]. Improvements from audit-and-feedback, however, often derive from motivating poor performing outliers rather than the average practitioner. Approaches that engage respected local clinical opinion-leaders may help to drive change, as these individuals may exert a disproportionate influence on their colleagues’ practice patterns [8,9]. Organizations should nurture and support these early adopters of practice change, and create opportunities for these physician-champions to share their experience with colleagues [4,8,9].

There is increasing enthusiasm among health care leaders for “tough love” policies that hold non-compliant clinicians accountable for following safe practices such as hand hygiene, use of central venous catheter placement bundles, and adoption of pre-operative checklists [10]. Given clear and convincing evidence that these practices reduce infection and improve outcomes, failure to follow such guidelines is regarded as reckless behavior and intolerable. However, guideline developers should not underestimate the challenge of incorporating apparently simple techniques into complex bedside activities.

Simple actions, like removing jewelry, wearing a surgical gown, or performing a hand scrub turn out to be highly sensitive to environmental factors and local social norms. Moving hand sanitizer within easy view and arms’ reach of a busy clinician can dramatically increase the rate of hand hygiene performance. Providing an easy place to secure jewelry or obtain a clean gown could also increase compliance. Similarly, the degree of collegiality on a clinical team, the culture of the unit, and the team leader’s willingness to model the behavior can drive practice more effectively than persistent exhortations. Practice is ultimately an activity that is performed by individuals on teams in units. Appreciating and addressing the way that clinicians practice at the bedside is essential to changing the way physicians work.

Adoption and spread of guidelines for neuraxial analgesia for labor in Israeli hospitals is good news for patients, but there is room for improvement. Guidelines are useful mechanisms for articulating safe practices in professional communities, but full, effective, and timely implementation requires careful attention to conditions on the ground in each hospital unit. Implementation is a more complex affair than we might expect. Those who promulgate national guidelines should remember that clinical practice is an inherently local activity, advancing by one unit, one team, and one clinician at a time.

## Competing interests

The author declares that he has no competing interests.

## Author’s information

Dr. Weingart is Chief Medical Officer and Senior Vice President for Medical Affairs at Tufts Medical Center, in Boston, Massachusetts.

## Commentary on

Ioscovich A, Davidson EM, Orbach-Zinger S, Rudich Z, Ivry S, Rosen LJ, Avidan A, Ginosar Y: Performance of Aseptic Technique During Neruaxial Analgesia for Labor Before and After the Publication of International Guidelines on Aseptic Technique. Isr J Health Policy Res 2014, 3:9
